# Quantifying knee cartilage development trajectories in children aged 6–12 years via diffusion tensor imaging

**DOI:** 10.3389/fped.2026.1833247

**Published:** 2026-07-01

**Authors:** Zhuo Cheng, Wei Li, Wei Ma, Gaohui Zhu, Yujuan Hu, Junya Ma, Sijie Gao, Yilu Zhang, Hailun Peng, Ye Xu

**Affiliations:** 1 Department of Radiology Children's Hospital of Chongqing Medical University, National Clinical Research Center for Children and Adolescents' Health and Diseases, Ministry of Education Key Laboratory of Child Development and Disorders, Chongqing Key Laboratory of Pediatric Metabolism and Inflammatory Diseases, Chongqing, China; 2 Department of Radiology, Yubei District People’s Hospital of Chongqing, Chongqing, China; 3 Department of Endocrinology Children's Hospital of Chongqing Medical University, National Clinical Research Center for Children and Adolescents' Health and Diseases, Ministry of Education Key Laboratory of Child Development and Disorders, Chongqing Key Laboratory of Pediatric Metabolism and Inflammatory Diseases, Chongqing, China

**Keywords:** cartilage, children, diffusion tensor imaging, magnetic resonance imaging, physical growth

## Abstract

**Objectives:**

This study aims to establish normative diffusion tensor imaging (DTI) biomarkers of pediatric knee cartilage development trajectories and sex-specific differences by quantifying microstructural changes in children aged 6–12 years.

**Methods:**

Eighty-four healthy children (43 boys and 41 girls; mean age 9.01 ± 1.84 years) underwent 3.0T MRI of the left knee, including DTI sequences (*b*-values: 0 and 600 s/mm^2^). Regions of interest included the growth plate, patellar, medial condylar, and lateral condylar cartilage. Fractional anisotropy (FA) (collagen integrity) and apparent diffusion coefficient (ADC) (proteoglycan/water content) were measured. Bone age was assessed via left-hand radiography according to the Chinese Wrist Bone Development Standard. The signal-to-noise ratio (SNR) of the DTI images was measured. Statistical analyses included Spearman correlation coefficients to assess age/bone age associations, intraclass correlation coefficients (ICCs) to evaluate reproducibility, and *t*-tests to compare sex/age group differences.

**Results:**

FA values increased significantly with age and bone age (*p* < 0.001), showing stronger correlations with bone age (boys: *r* = 0.843; girls: *r* = 0.789) than with chronological age. Girls exhibited higher FA values than boys across all age groups (*p* < 0.05), particularly within the growth plate cartilage. ADC values in the growth plate decreased with increasing bone age (girls: *r* = −0.702; boys: *r* = −0.511; *p* < 0.001), with a steeper decline observed in girls. No significant correlations between ADC and age were found in other regions. SNR correlated positively with age and bone age, with ICC confirming excellent reproducibility (FA/ADC ICC > 0.94).

**Conclusion:**

DTI biomarkers (FA/ADC) sensitively reflect pediatric knee cartilage maturation, correlate best with bone age, and enhance quantitative monitoring of cartilage development and pathology in children.

## 1 Introduction

Developing cartilage in children comprises three distinct regions: growth plate cartilage (GPC), epiphyseal cartilage, and articular cartilage. While these regions share the same extracellular matrix, composed mainly of collagen fibrils and polymeric proteoglycans (PGs), they differ in their structural organization (1–3). Specifically, longitudinal bone growth occurs within the growth plate, epiphyseal cartilage facilitates circumferential bone enlargement, and articular cartilage enables smooth joint motion (1, 4–7).

Studying developing cartilage in children has significant clinical value, as it establishes quantitative developmental characteristics that address limitations in X-ray bone age accuracy. Early detection of pathological cartilage changes is crucial for preventing irreversible growth retardation and skeletal deformities (2, 8, 9). Magnetic resonance (MR) diffusion tensor imaging (DTI) has recently enabled quantitative assessment of cartilage, primarily in adult articular cartilage. Fractional anisotropy (FA) provides information on collagen structural integrity, while the apparent diffusion coefficient (ADC) reflects changes in PG and water content (10). A limited number of studies have applied DTI and fiber tractography to pediatric growth plate cartilage to evaluate growth potential (6). Collectively, these findings indicate that DTI measurements can serve as biomarkers of cartilage microstructure, development, and damage (11, 12). However, research on pediatric DTI cartilage remains limited and predominantly involves participants older than 12 years. Only one published study has investigated the development of epiphyseal and articular cartilage using DTI; this study did not analyze the distinct regions of epiphyseal cartilage and reported findings that conflicted with prior literature (13). The age range of 6–12 represents a peak period for cartilage development. During this stage, cartilage is highly sensitive to the effect of disease, warranting an in-depth study (14–16).

Therefore, this study aims to recruit a cohort of healthy children aged 6–12 years. Using MR DTI, we measured FA and ADC values within the knee cartilage. Analysis included elucidating age-, bone age-, and sex-dependent trends to characterize cartilage developmental patterns and establish normative reference ranges for these DTI parameters.

## 2 Materials and methods

### 2.1 Participants

**Figure 1:**
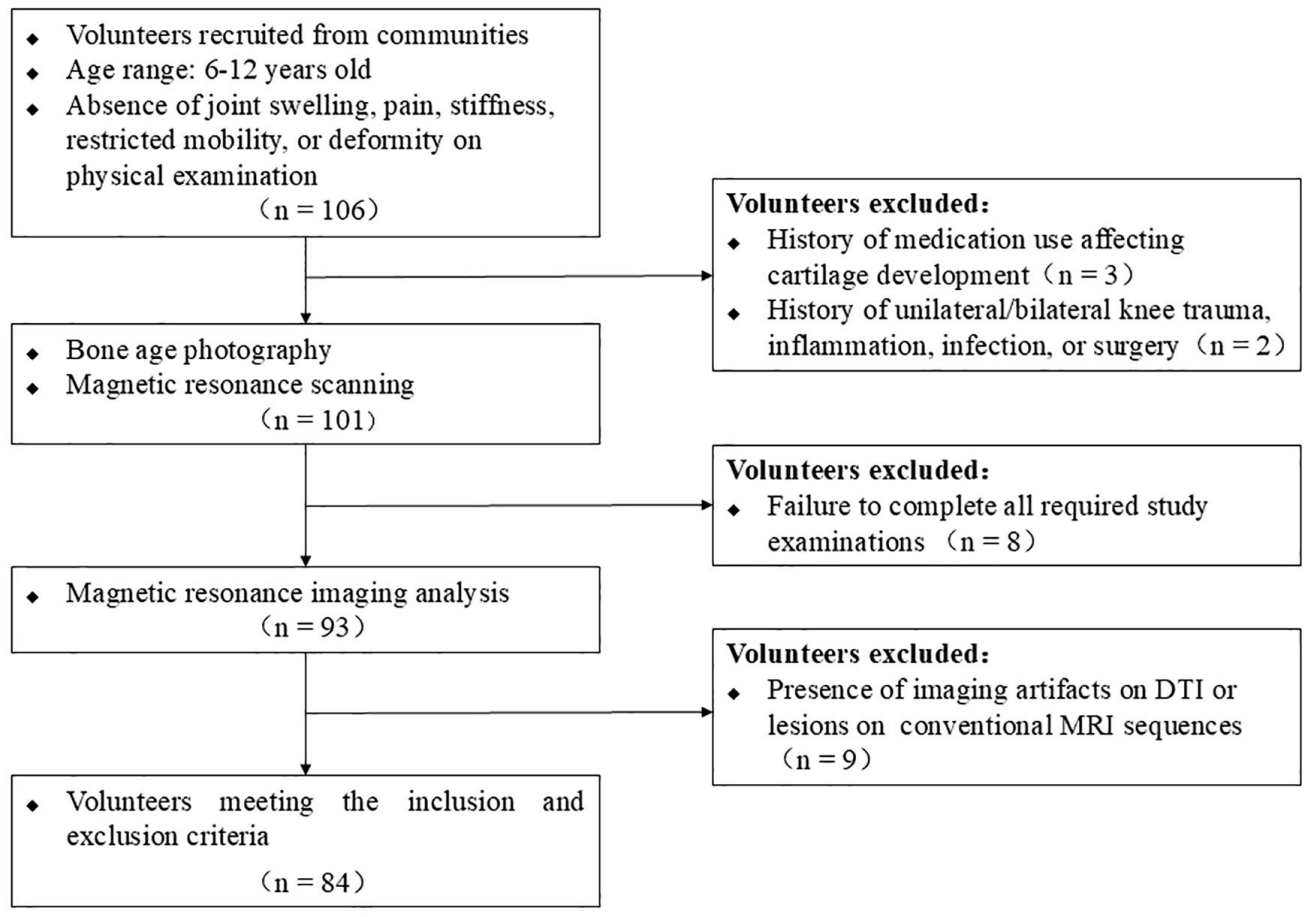
Flowchart of the study population.

This study was approved by the Medical Ethics Committee of our institution [Approval No. 2021 Ethical Review (Yan) No. 222]. Written informed consent was obtained from all volunteers and their guardians. Healthy children were recruited from communities in Chongqing, China, between May and July 2023. Participants were eligible for inclusion if they (1) were aged 6–12 years; and (2) had no evidence of joint abnormalities (swelling, pain, stiffness, restricted movement, or deformity, etc.), as confirmed by a comprehensive physical examination. Exclusion criteria were as follows: (1) inability to complete all examinations (e.g., due to claustrophobia); (2) presence of genetic metabolic diseases, immune disorders, tumors, or other multisystem diseases; (3) history of unilateral/bilateral knee trauma, inflammation, infection, or surgery; (4) prior use of glucocorticoids, growth hormones, thyroid hormones, or other cartilage-impacting medications; (5) participation in prolonged high-intensity exercise training; (6) consumption of royal jelly, bovine colostrum, vitamin AD, or similar health products for ≥7 days per year; and (7) significant artifacts in the left knee joint image or evident lesions on conventional MR imaging sequences. The inclusion and exclusion process is detailed in Figure 1.

### 2.2 Bone age measurement

Left-hand radiographs were acquired using a GE Discovery XR656 digital radiography (DR) system (parameters: 50 kV, 1.2 mAs, and 100 cm distance). Two experienced radiologists (W.M., S.G.; each with more than 5 years of experience in pediatric radiology) independently assessed the left-hand DR images. The real age information of the volunteers was concealed, while their gender information was retained. Bone age was determined according to the Chinese Wrist Bone Development Standard (CHN) method (17), and the mean of both assessments was recorded.

### 2.3 Magnetic resonance imaging

Volunteers remained seated for ∼5 min prior to scanning to allow the left knee to relax. Imaging was performed using a GE Discovery MR750 3.0-T scanner equipped with an eight-channel knee coil, acquiring conventional and DTI sequences. Imaging parameters are listed in Table 1.

**Table 1:** Details of the MR imaging protocol.

Parameter	T1WI	PDWI	DTI
TE/TR (ms)	5.1/400	35/2,200	56.5/2,000
NEX	2	2	2
FOV (mm)	160 × 160	160 × 160	160 × 160
Matrix size	288 × 224	288 × 224	80 × 80
Slice thickness/gap (mm)	3.5/0.5	3.5/0.5	3.5/0.5
Number of slices	15	15	15
Spatial resolution (mm)	0.6 × 0.7 × 3.5	0.6 × 0.7 × 3.5	2 × 2 × 3.5
Readout bandwidth (Hz/Px)	83.33	50	250
Diffusion gradient direction	…	…	25
*b*-Value	…	…	0/600
Scan time (s)	92	120	106

T1WI, T1-weighted imaging; PDWI, proton density-weighted imaging; DTI, diffusion tensor imaging; TE, echo time; TR, repetition time; NEX, number of excitation; FOV, field of view.

### 2.4 DTI image analysis

**Figure 2:**
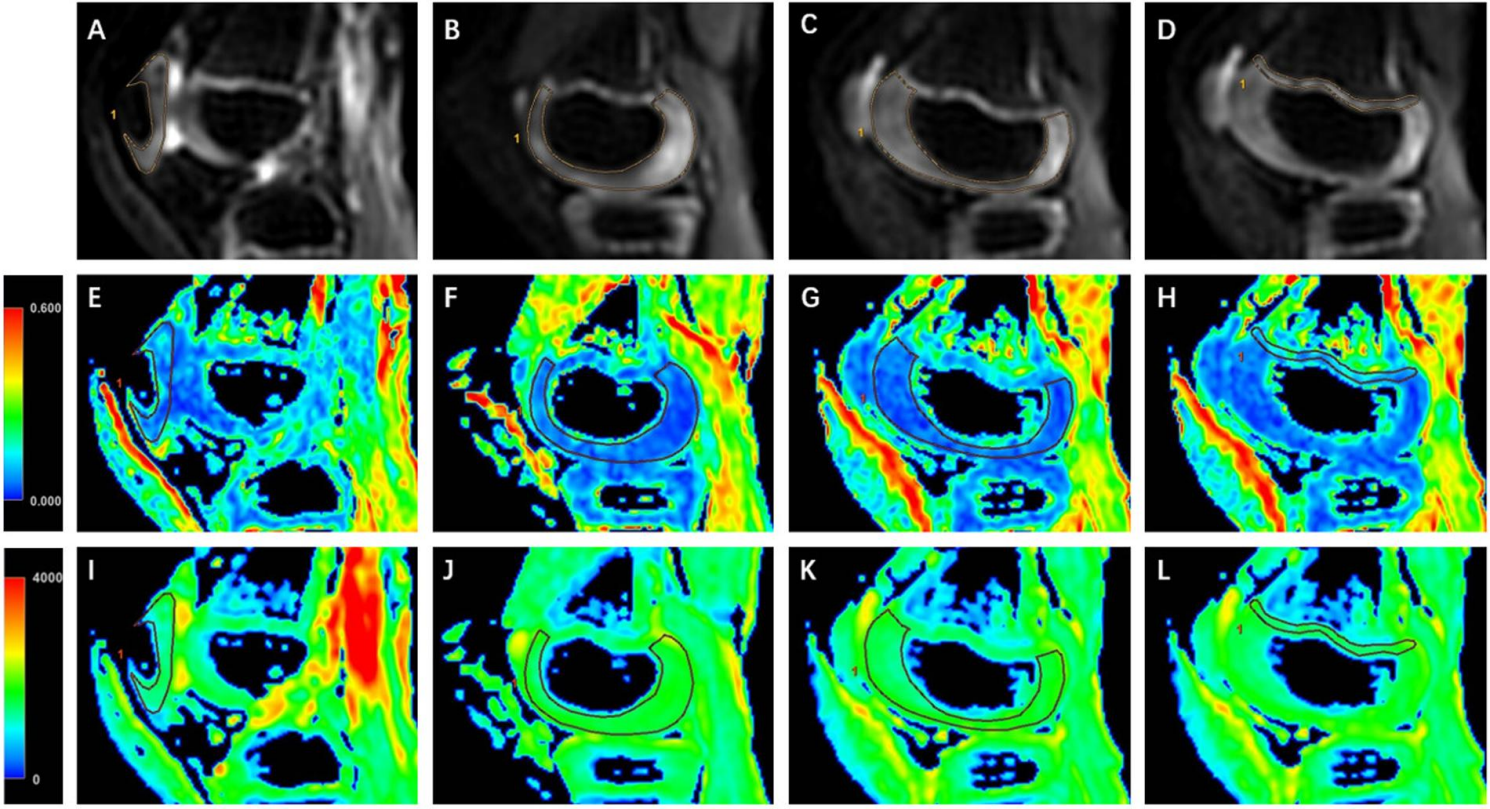
Representative FA and ADC measurements across four ROIs in the left knee joint. **(A–D)** Anatomical localization of ROIs on *b* = 0 s/mm^2^ images: **(A)** Patellar cartilage ROI (encompassing articular and epiphyseal cartilage). **(B)** Medial femoral condylar cartilage ROI (including the articular cartilage and epiphyseal cartilage of the medial femoral condyle). **(C)** Lateral femoral condylar cartilage ROI (including the articular cartilage and epiphyseal cartilage of the lateral femoral condyle). **(D)** Growth plate cartilage ROI. **(E–H)** FA maps corresponding to the ROIs in **(A–D)**, with values automatically generated by postprocessing software. **(I–L)** ADC maps corresponding to the ROIs in **(A–D)**, with values automatically computed by postprocessing software.

DTI datasets were transferred to a GE ADW 4.7 workstation. Using ReadyView DTI software, images were automatically registered, and planar echo images (*b* = 0 s/mm^2^), FA pseudocolor maps, and ADC pseudocolor maps were generated. Two radiologists (Z.C., H.P., each with more than 5 years of experience in pediatric MRI) selected the images that best displayed the morphology and boundaries of the cartilage on the planar echo images and manually delineated four regions of interest (ROIs)—patellar cartilage, medial femoral condyle cartilage, lateral femoral condyle cartilage, and femoral growth plate cartilage. These ROIs were mapped onto the FA and ADC maps for automatic calculation. The delineation methodology followed our previous report and is shown in Figure 2 (18). Each radiologist performed three measurements per ROI, yielding six measurements per region. The mean of these six measurements represented the FA/ADC value for that ROI. The overall knee cartilage FA/ADC value was calculated as the mean across all four ROIs. Calculation used the standard formulas:

$$\mathrm{FA}=\frac{1}{\sqrt{2}}\sqrt{\frac{{\left(\lambda 1-\lambda 2\right)}^{2}+{\left(\lambda 2-\lambda 3\right)}^{2}+{\left(\lambda 1-\lambda 3\right)}^{2}}{\lambda 1^{2}+\lambda 2^{2}+\lambda 3^{2}}}$$

$$\mathrm{ADC}=\frac{\lambda 1+\lambda 2+\lambda 3}{3}$$

where *λ*1, *λ*2, and *λ*3 represent the three eigenvalues of the diffusion tensor (19).

### 2.5 Signal-to-noise ratio evaluation

**Figure 3:**
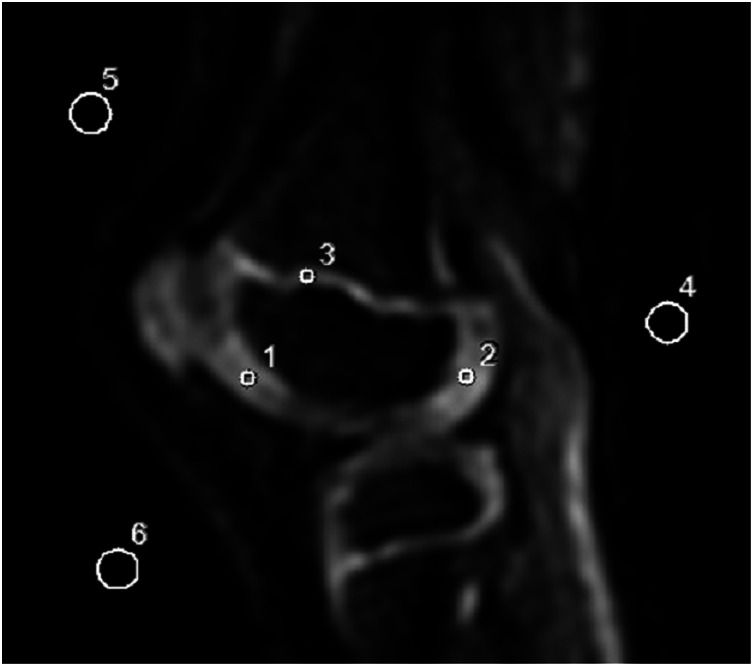
Representative ROI placement for SNR calculation on *b* = 600 s/mm^2^ images. ROIs for cartilage signal intensity: positioned at the midpoint of the (1) anterior articular and epiphyseal cartilage of lateral femoral condylar cartilage, (2) posterior articular and epiphyseal cartilage of lateral femoral condylar cartilage, and (3) growth plate cartilage. ROIs for background noise standard deviation: located (4) in the popliteal fossa, (5) anterior to the patella, and (6) anterior to the tibia.

To ensure data stability, the signal-to-noise ratio (SNR) was measured on DTI raw images (*b* = 600 s/mm^2^) using a GE ADW 4.7 workstation. For each participant, one image clearly displaying the trochlear articular cartilage, the lateral femoral condyle cartilage, and the growth plate cartilage was selected. ROIs were delineated within these three cartilage regions (each ROI with an area of 0.3 cm^2^), and their mean signal intensity (SI) was calculated. Background noise was estimated using three ROIs placed outside the knee joint (each ROI with an area of 3.0 cm^2^). Image SNR was calculated as follows: SNR = 0.655 × (mean cartilage SI)/(mean standard deviation of background SI) (20). The method is detailed in Figure 3.

### 2.6 Intra- and interobserver variability

Three months after the initial analysis, ROIs were redelineated for a random subset of 30 volunteers by the same observers (Z.C., H.P.), who were blinded to the prior measurements, for patellar, medial condyle, lateral condyle, and growth plate cartilage. Bone age and the corresponding cartilage FA/ADC values were compared to assess variability.

### 2.7 Statistical analysis

Data were analyzed using IBM SPSS Statistics 26.0. The Shapiro–Wilk test was used for normality testing. If the data were normally distributed, they were expressed as mean ± standard deviation; otherwise, the data were expressed as median (quartiles). Intraclass correlation coefficients (ICCs; two-way mixed-effects model, absolute agreement) were used to assess intra- and interobserver variability. Spearman correlation was used to evaluate associations between (1) age and bone age; (2) image SNR and age and bone age; and (3) cartilage FA/ADC values and age and bone age. Independent-samples *t*-tests were used to compare FA/ADC values between different age groups, bone age groups, and genders. A two-sided *p*-value <0.05 was considered statistically significant.

## 3 Results

### 3.1 Study population

Eighty-four healthy children (43 boys and 41 girls; mean age 9.01 ± 1.84 years, range 6–12) were enrolled. The mean bone age was 9.86 ± 2.19 (range 5.7–14.0) and showed a strong correlation with chronological age (*r* = 0.875, *p* < 0.001). Participants were stratified into (1) age groups: 6–8 years (14 boys, 15 girls), 8–10 years (12 boys, 13 girls), 10–12 years (17 boys, 13 girls); and (2) bone age groups: 5–8 years (eight boys, 11 girls), 8–10 years (11 boys, 11 girls), 10–12 years (15 boys, 10 girls), and 12–14 years (nine boys, nine girls) (Table 2).

**Table 2:** General information of the study population.

Group	Group 1	Group 2	Group 3	Group 4
Age group				
Total no.	29	25	30	…
Age range (years)	6–8	8–10	10–12	…
No. of boys	14	12	17	…
Mean age (years)^a^	6.9 ± 0.6	8.9 ± 0.6	11.0 ± 0.5	…
No. of girls	15	13	13	…
Mean age (years)^a^	7.0 ± 0.6	8.8 ± 0.7	11.2 ± 0.7	…
Bone age group				
Total no.	19	22	25	18
Bone age range (years)	5–8	8–10	10–12	12–14
No. of boys	8	11	15	9
Mean bone age (years)^a^	6.9 ± 0.7	9.3 ± 0.5	10.8 ± 0.7	13.0 ± 0.8
No. of girls	11	11	10	9
Mean bone age (years)^a^	6.7 ± 0.8	8.9 ± 0.4	10.8 ± 0.4	12.7 ± 0.6

a Data are presented as mean ± SD.

### 3.2 Reproducibility and image quality

ICC analysis demonstrated excellent measurement reproducibility. For bone age assessment, the intraobserver ICC was 0.98 (95% CI: 0.96–0.99, *p* < 0.001). For cartilage microstructural metrics, the intraobserver ICC was 0.96 (95% CI: 0.94–0.98, *p* < 0.001) for both FA and ADC values.

**Figure 4:**
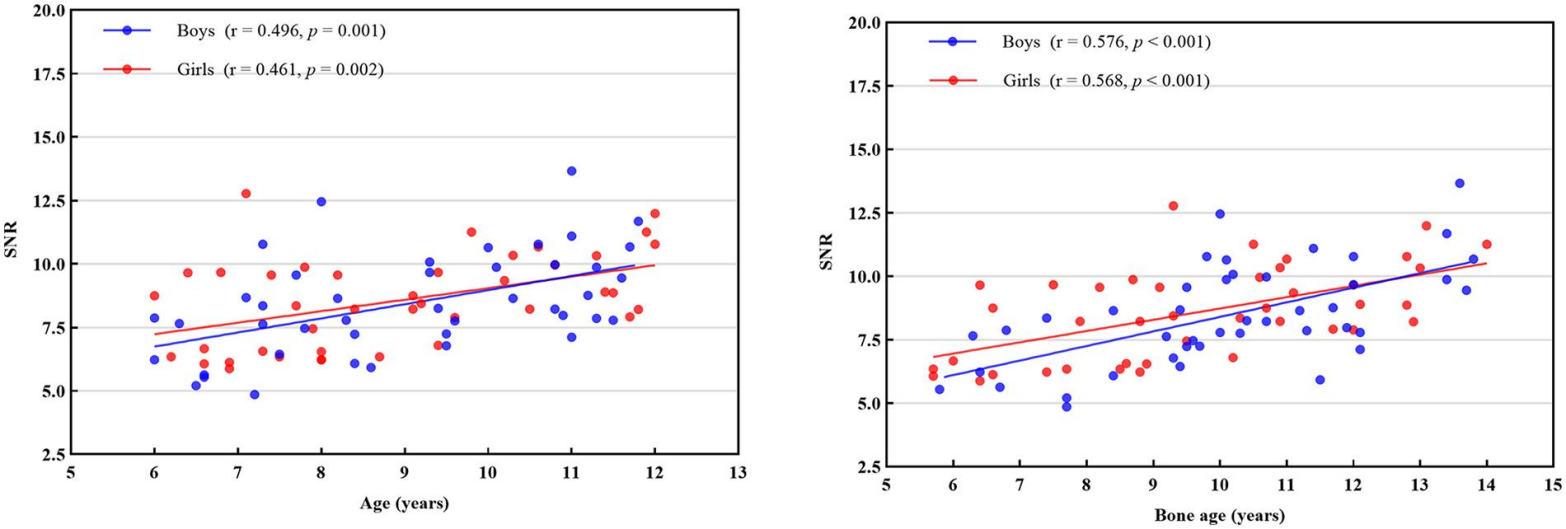
Correlation between SNR and developmental indices (age and bone age). All participants exhibited SNR values ≥4.86. SNR demonstrated positive correlations with both chronological age and bone age, with a stronger correlation observed for bone age.

Analysis of images acquired at *b* = 600 s/mm^2^ revealed a mean SNR of 8.23 (range 4.86–13.66) in boys, which showed significant positive correlations with both chronological age (*r* = 0.496, *p* = 0.001) and bone age (*r* = 0.576, *p* < 0.001). In girls, the mean SNR was 8.45 (range 5.87–12.78), and it also showed significant positive correlations with both chronological age (*r* = 0.461, *p* = 0.002) and bone age (*r* = 0.568, *p* < 0.001) (Table 3, Figure 4).

**Table 3:** Correlation of image SNR with age and bone age.

	SNR	Age		Bone age	
Gender		*r*	*p*	*r*	*p*
Boys	8.23 (4.86–13.66)	0.496	0.001	0.576	<0.001
Girls	8.45 (5.87–12.78)	0.461	0.002	0.568	<0.001

SNR values are presented as medians, with ranges in parentheses.

### 3.3 Association between cartilage microstructure and age and bone age

**Figure 5:**
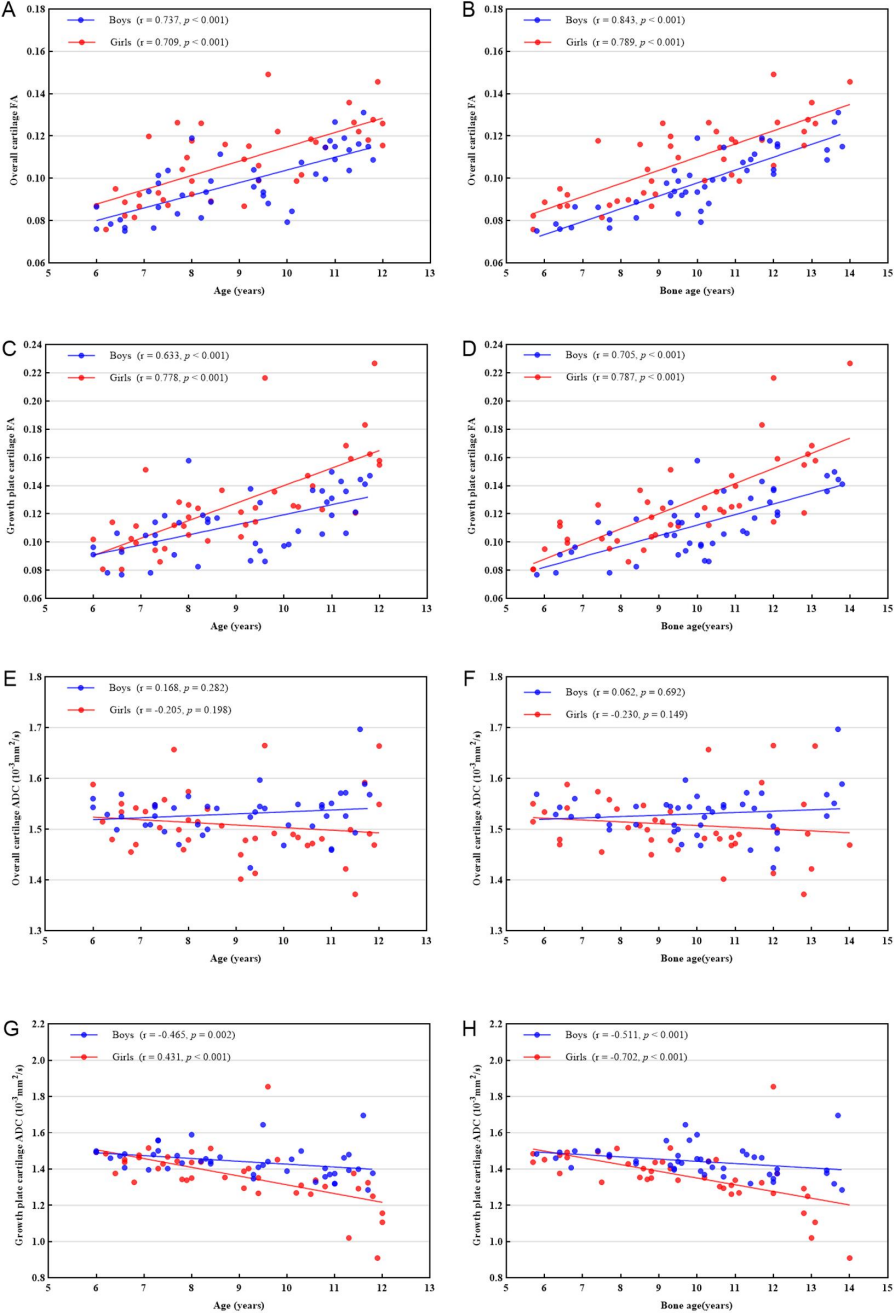
Scatter plots depicting associations between FA/ADC values in overall knee cartilage/growth plate cartilage and developmental indices (age, bone age) stratified by gender. **(A,B)** Overall cartilage FA: significant positive correlations were observed with both chronological age and bone age, with bone age exhibiting a stronger correlation. Correlation slopes were steeper in boys than in girls for both age and bone age. **(C,D)** Growth plate cartilage FA: positive correlations were observed with age and bone age, although bone age correlation remained superior. Girls demonstrated significantly stronger correlations than boys for both parameters. **(E,F)** Overall cartilage ADC: no significant correlation was observed with chronological age or bone age. **(G,H)** Growth plate cartilage ADC: significant negative correlations were observed with both age and bone age, with bone age exhibiting a stronger correlation. Girls showed steeper regression slopes than boys for both age and bone age.

Spearman analysis showed significant positive correlations between FA values (overall and regional) and both age and bone age (*p* < 0.05). Specifically, for overall FA, the correlations were stronger with bone age (boys *r* = 0.843, girls *r* = 0.789) than with chronological age (boys *r* = 0.737; girls *r* = 0.709). Boys exhibited stronger age and bone age correlations than girls. For regional FA, growth plate FA in girls was more strongly correlated with age and bone age (*r* ≥ 0.778) than in boys (*r* ≥ 0.633) (*p* < 0.001). For ADC trends, growth plate ADC decreased with age and bone age, with stronger correlations observed for bone age (boys *r* = −0.511, girls *r* = −0.702) than for age (boys *r* = −0.465, girls *r* = −0.692). Girls showed stronger correlations than boys (*p* < 0.001). No significant correlations were observed for ADC in the other regions (Table 4, Figure 5).

**Table 4:** Correlation of FA and ADC values with age and bone age.

	Boys				Girls			
	Age		Bone age		Age		Bone age	
Cartilage partition	*r*	*p*	*r*	*p*	*r*	*p*	*r*	*p*
FA value								
OC	0.737	<0.001*	0.843	<0.001*	0.709	<0.001*	0.789	<0.001*
PC	0.699	<0.001*	0.773	<0.001*	0.600	<0.001*	0.647	<0.001*
MCC	0.388	0.010*	0.431	0.004*	0.369	0.018*	0.473	0.002*
LCC	0.563	<0.001*	0.693	<0.001*	0.639	<0.001*	0.702	<0.001*
GPC	0.633	<0.001*	0.705	<0.001*	0.778	<0.001*	0.787	<0.001*
ADC value								
OC	0.168	0.282	0.062	0.692	−0.205	0.198	−0.230	0.149
PC	0.129	0.410	0.100	0.524	0.117	0.465	0.139	0.386
MCC	0.242	0.118	0.123	0.433	0.228	0.152	0.242	0.128
LCC	0.307	0.045*	0.175	0.262	−0.049	0.761	−0.070	0.665
GPC	−0.465	0.002	−0.511	<0.001*	−0.692	<0.001*	−0.702	<0.001*

OC, overall cartilage; PC, patellar cartilage; MCC, medial condylar cartilage; LCC, lateral condylar cartilage; GPC, growth plate cartilage.

* *p* < 0.05.

### 3.4 Sex-related differences in cartilage microstructure

**Figure 6:**
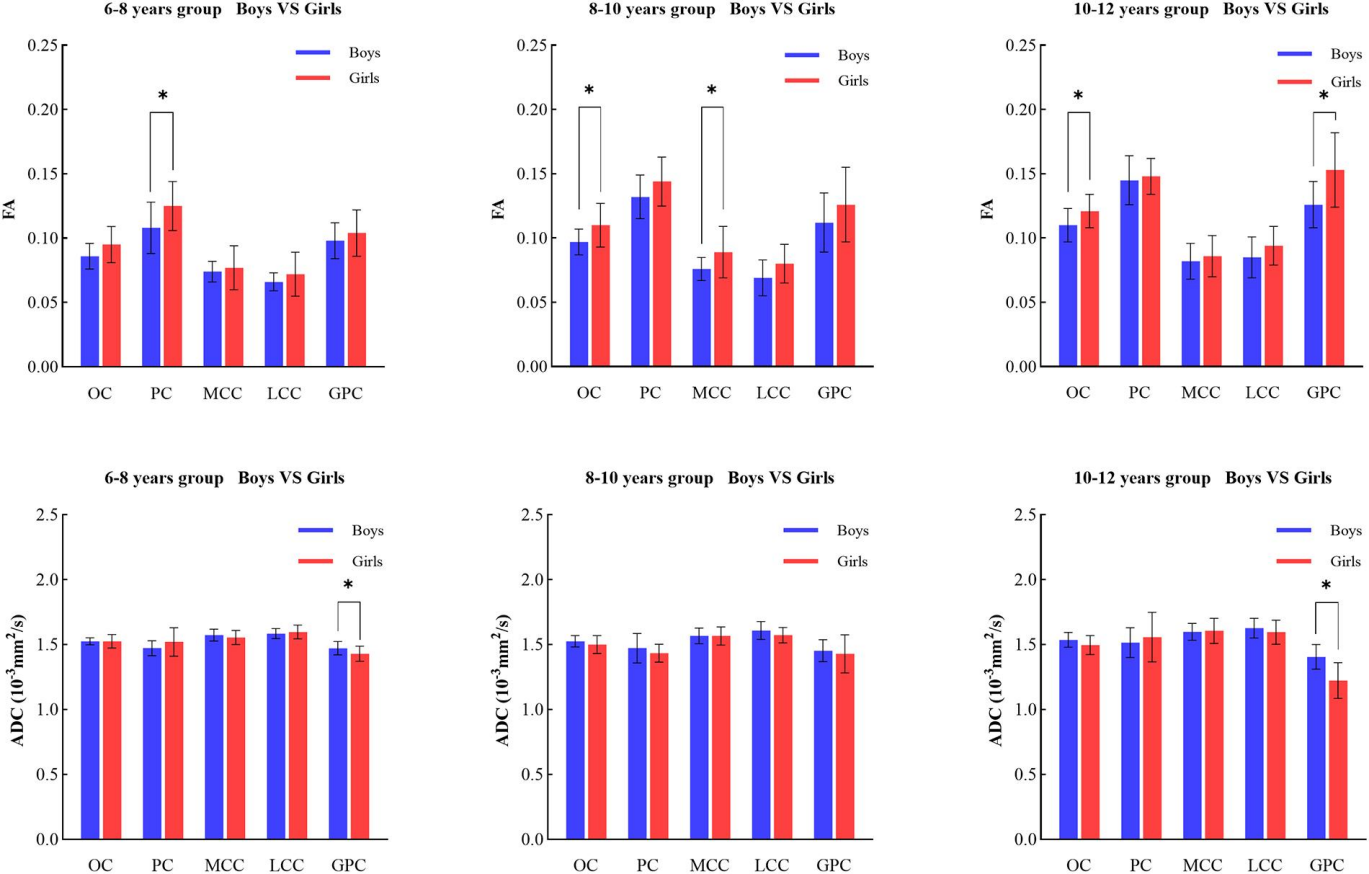
Sex-based comparison of FA and ADC values in knee cartilage subregions across age groups. OC, overall cartilage; PC, patellar cartilage; MCC, medial condylar cartilage; LCC, lateral condylar cartilage; GPC, growth plate cartilage; *p < 0.05.

Age-stratified analysis revealed significantly lower FA values in boys than in girls across both overall and regional knee cartilage assessments. Statistically significant sex differences (*p* < 0.05) were observed in specific age cohorts: patellar cartilage FA in the 6–8-year group (*p* = 0.023), overall FA (*p* = 0.039) and medial condylar cartilage FA (*p* = 0.047) in the 8–10-year group, and overall FA (*p* = 0.029) and growth plate cartilage FA (*p* = 0.004) in the 10–12-year group (Table 5, Figure 6). For ADC values, boys exhibited higher growth plate ADC values in both the 6–8-year (*p* = 0.048) and 10–12-year cohorts (*p* < 0.001). Notably, patellar and condylar ADC values consistently exceeded growth plate ADC values across all age groups, indicating region-specific microstructural properties independent of sex.

**Table 5:** Intersexual comparison of knee cartilage FA and ADC values in different age groups.

	6–8 (years)				8–10 (years)				10–12 (years)			
Cartilage partition	Boys (*n* = 14)	Girls (*n* = 15)	*t*	*p*	Boys (*n* = 12)	Girls (*n* = 13)	*t*	*p*	Boys (*n* = 17)	Girls (*n* = 13)	*t*	*p*
FA value												
OC	0.086 ± 0.010	0.095 ± 0.014	−1.814	0.081	0.097 ± 0.010	0.110 ± 0.017	−2.194	0.039*	0.110 ± 0.013	0.121 ± 0.013	−2.309	0.029*
PC	0.108 ± 0.020	0.125 ± 0.019	−2.414	0.023*	0.132 ± 0.017	0.144 ± 0.019	−1.608	0.122	0.145 ± 0.019	0.148 ± 0.014	−0.502	0.620
MCC	0.074 ± 0.008	0.077 ± 0.017	−0.710	0.484	0.076 ± 0.009	0.089 ± 0.020	−2.098	0.047*	0.082 ± 0.014	0.086 ± 0.016	−0.846	0.405
LCC	0.066 ± 0.007	0.072 ± 0.017	−1.210	0.237	0.069 ± 0.014	0.080 ± 0.015	−1.942	0.065	0.085 ± 0.016	0.094 ± 0.015	−1.604	0.120
GPC	0.098 ± 0.014	0.104 ± 0.018	−1.097	0.282	0.112 ± 0.023	0.126 ± 0.029	−1.370	0.184	0.126 ± 0.018	0.153 ± 0.029	−3.112	0.004*
ADC value												
OC	1.526 ± 0.027	1.526 ± 0.053	0.001	0.999	1.526 ± 0.043	1.501 ± 0.068	1.080	0.291	1.537 ± 0.057	1.496 ± 0.073	1.725	0.096
PC	1.473 ± 0.058	1.521 ± 0.109	−1.465	0.155	1.473 ± 0.114	1.434 ± 0.069	1.038	0.310	1.516 ± 0.114	1.558 ± 0.190	−0.752	0.458
MCC	1.574 ± 0.046	1.556 ± 0.054	0.959	0.346	1.568 ± 0.061	1.567 ± 0.070	0.005	0.996	1.599 ± 0.064	1.607 ± 0.097	−0.275	0.786
LCC	1.585 ± 0.038	1.597 ± 0.053	−0.721	0.477	1.609 ± 0.069	1.573 ± 0.060	1.407	0.173	1.628 ± 0.076	1.596 ± 0.092	1.040	0.307
GPC	1.472 ± 0.051	1.430 ± 0.059	2.070	0.048*	1.454 ± 0.084	1.430 ± 0.147	0.497	0.624	1.406 ± 0.095	1.224 ± 0.138	4.280	<0.001*

Data are presented as mean ± SD. OC, overall cartilage; PC, patellar cartilage; MCC, medial condylar cartilage; LCC, lateral condylar cartilage; GPC, growth plate cartilage. ADC values are in units of ×10⁻^3^ mm^2^/s.

* *p* < 0.05.

**Figure 7:**
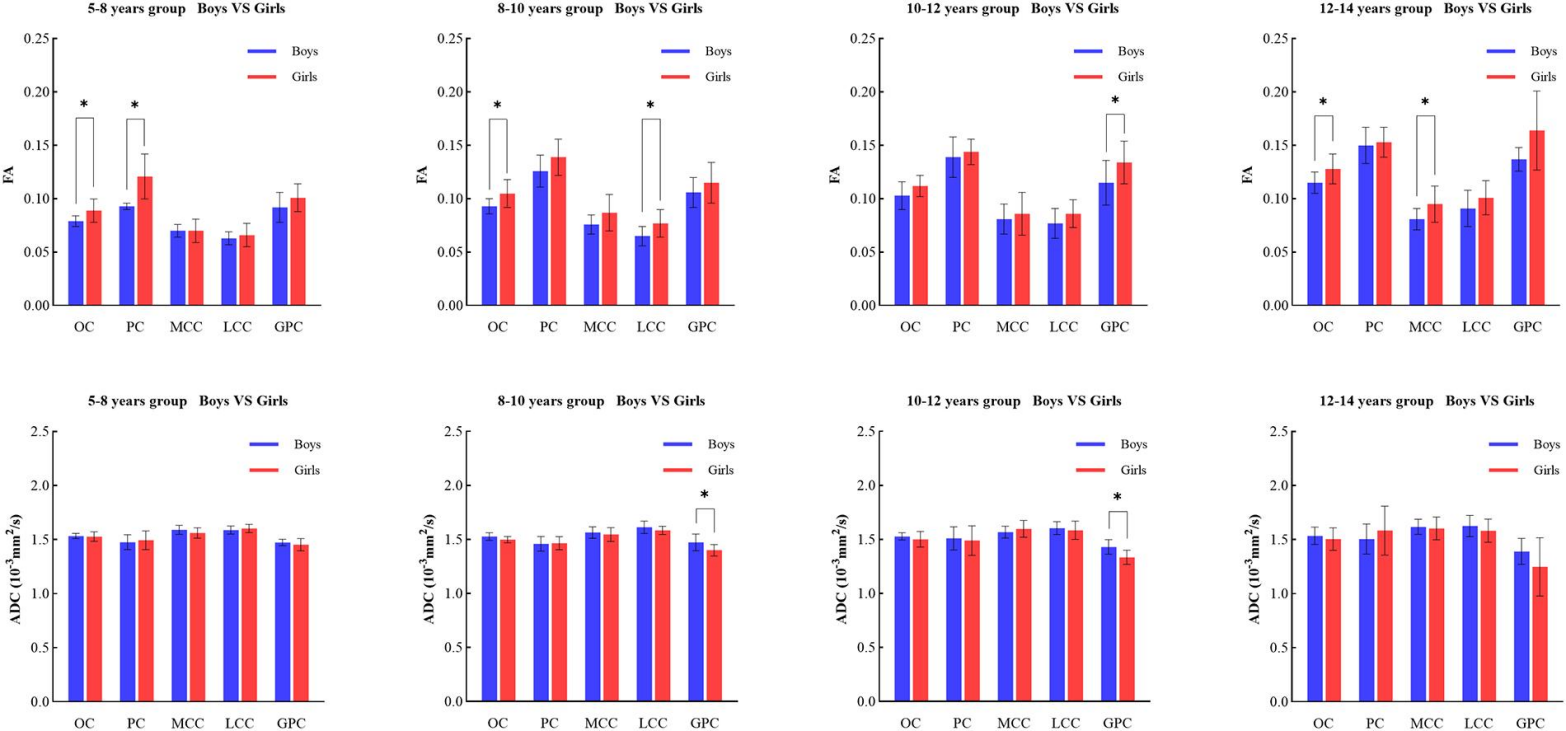
Sex-based comparison of FA and ADC values in knee cartilage subregions across bone age groups. OC, overall cartilage; PC, patellar cartilage; MCC, medial condylar cartilage; LCC, lateral condylar cartilage; GPC, growth plate cartilage; *p < 0.05.

Bone age-stratified analysis further corroborated these patterns. Boys demonstrated significantly reduced overall FA values in the 5–8-year (*p* = 0.026), 8–10-year (*p* = 0.021), and 12–14-year groups (*p* = 0.027). Regionally, FA sex differences were prominent in the patellar cartilage (5–8 years, *p* = 0.002), lateral condylar (8–10 years, *p* = 0.015), growth plate (10–12 years, *p* = 0.034), and medial condyle (12–14 years, *p* = 0.049). Growth plate ADC remained higher in boys within the 8–10-year (*p* = 0.020) and 10–12-year bone age groups (*p* = 0.002). Similar to chronological age stratification, patellar and condylar ADC values were generally higher than the growth plate ADC values, except in the 8–10-year cohort, where this pattern reversed (Table 6, Figure 7).

**Table 6:** Intersexual comparison of knee cartilage FA and ADC values in different bone age groups.

	5–8 (years)				8–10 (years)				10–12 (years)				12–14 (years)			
Cartilage partition	Boys (*n* = 8)	Girls (*n* = 11)	*t*	*p*	Boys (*n* = 11)	Girls (*n* = 11)	*t*	*p*	Boys (*n* = 15)	Girls (*n* = 10)	*t*	*p*	Boys (*n* = 9)	Girls (*n* = 9)	*t*	*p*
FA value																
OC	0.079 ± 0.005	0.089 ± 0.011	−2.435	0.026*	0.093 ± 0.007	0.105 ± 0.013	−2.505	0.021*	0.103 ± 0.013	0.112 ± 0.010	−2.025	0.055	0.115 ± 0.010	0.128 ± 0.014	−2.441	0.027*
PC	0.093 ± 0.003	0.121 ± 0.021	−3.711	0.002*	0.126 ± 0.015	0.139 ± 0.017	−1.860	0.078	0.139 ± 0.019	0.144 ± 0.012	−0.711	0.484	0.150 ± 0.017	0.153 ± 0.014	−0.447	0.661
MCC	0.070 ± 0.006	0.070 ± 0.011	−0.006	0.995	0.076 ± 0.009	0.087 ± 0.017	−1.967	0.063	0.081 ± 0.014	0.086 ± 0.020	−0.823	0.419	0.081 ± 0.010	0.095 ± 0.017	−2.135	0.049*
LCC	0.063 ± 0.006	0.066 ± 0.011	−0.538	0.598	0.065 ± 0.009	0.077 ± 0.013	−2.673	0.015*	0.077 ± 0.014	0.086 ± 0.013	−1.752	0.093	0.091 ± 0.017	0.101 ± 0.016	−1.256	0.227
GPC	0.092 ± 0.014	0.101 ± 0.013	−1.423	0.173	0.106 ± 0.014	0.115 ± 0.019	−1.343	0.194	0.115 ± 0.021	0.134 ± 0.020	−2.256	0.034*	0.137 ± 0.011	0.164 ± 0.037	−2.121	0.050
ADC value																
OC	1.533 ± 0.024	1.528 ± 0.043	0.276	0.786	1.528 ± 0.35	1.499 ± 0.030	2.041	0.055	1.529 ± 0.035	1.502 ± 0.071	1.275	0.215	1.535 ± 0.080	1.505 ± 0.105	0.687	0.502
PC	1.476 ± 0.069	1.494 ± 0.087	−0.485	0.634	1.460 ± 0.068	1.466 ± 0.061	−0.221	0.827	1.511 ± 0.107	1.490 ± 0.136	0.433	0.669	1.505 ± 0.139	1.584 ± 0.226	−0.902	0.380
MCC	1.591 ± 0.042	1.562 ± 0.048	1.378	0.186	1.565 ± 0.053	1.546 ± 0.064	0.736	0.470	1.568 ± 0.055	1.598 ± 0.077	−1.151	0.262	1.618 ± 0.070	1.604 ± 0.106	0.332	0.745
LCC	1.589 ± 0.038	1.603 ± 0.038	−0.793	0.439	1.613 ± 0.057	1.584 ± 0.037	1.374	0.185	1.606 ± 0.060	1.585 ± 0.084	0.746	0.463	1.626 ± 0.098	1.583 ± 0.108	0.870	0.397
GPC	1.474 ± 0.030	1.453 ± 0.056	0.965	0.348	1.473 ± 0.079	1.400 ± 0.054	2.529	0.020*	1.431 ± 0.067	1.335 ± 0.066	3.526	0.002*	1.391 ± 0.120	1.248 ± 0.270	1.454	0.165

Data are presented as mean ± SD. OC, overall cartilage; PC, patellar cartilage; MCC, medial condylar cartilage; LCC, lateral condylar cartilage; GPC, growth plate cartilage. ADC values are in units of ×10⁻^3^ mm^2^/s.

* *p* < 0.05.

## 4 Discussion

The microstructural development of pediatric knee cartilage follows distinct maturational patterns that can be quantified using MR DTI. This study demonstrates significant correlations between DTI-derived biomarkers (FA and ADC values) and key developmental indices—bone age and sex. Notably, in both boys and girls, FA and ADC values exhibited stronger correlations with bone age than with chronological age, reinforcing bone age as a superior indicator of biological maturity that more accurately reflects physical development trajectories (21–23).

Our findings reveal progressive increases in FA across all knee cartilage regions with advancing age and bone age. This aligns with prior observations by Jaimes et al. and Kvist et al., supporting the role of FA in tracking collagen matrix organization (7, 24). Mechanistically, FA quantifies water diffusion anisotropy, which is determined by tissue microstructure rather than composition (25–27). During development, cartilage thinning coincides with chondrocyte proliferation and collagen densification, enhancing extracellular matrix organization. This structural refinement restricts water diffusion perpendicular to collagen fibers, thereby increasing FA values (28).

Sex-specific differences further highlight the sensitivity of FA, as girls consistently exhibited higher FA values than boys within matched age and bone age groups. We propose that this finding reflects earlier cartilage maturation in girls, characterized by accelerated collagen alignment and cellular organization (29). This finding, previously unreported, underscores sex as a critical covariate in developmental cartilage imaging.

In contrast to FA, ADC values displayed region-specific patterns. Patellar, medial, and lateral condylar cartilage ADC values exceeded those of growth plate cartilage in most age groups—except patellar ADC in the 8–10-year bone-age cohort. This observation corroborates porcine femur data reported by Jaramillo et al. (30) and may be attributed to three factors: (1) Structural divergence: Growth plate cartilage exhibits strong longitudinal fiber orientation, limiting isotropic diffusion (lower ADC). In contrast, epiphyseal cartilage, which governs transverse expansion, permits greater water mobility (higher ADC) (31). (2) Maturational changes: Ossification reduces proteoglycan content while increasing chondrocyte dispersion and free water content, thereby elevating epiphyseal ADC (31, 32). (3) Partial volume effects: Our epiphyseal ROIs included outer articular layers with inherently weaker diffusion anisotropy. Critically, growth plate ADC decreased with age and bone age, correlating more strongly with bone age (*r* = −0.702 in girls) than with chronological age. This decline reflects growth plate thinning, reduced extracellular space, and decreased water content during puberty (7, 33, 34). Girls demonstrated steeper ADC declines than boys, which aligns with their earlier maturation onset.

Methodologically, this study optimized DTI protocols to enhance reliability: (1) Sagittal imaging improved visualization of longitudinal collagen alignment compared with axial and coronal planes (18). (2) Previous studies (35, 36) and preliminary experiments carried out by our team indicated that a low signal-to-noise ratio often results in overestimated FA values. In this study, the signal-to-noise ratio was enhanced (SNR > 4.86) by reducing the matrix size (80 × 80), thereby minimizing the risk of FA overestimation. The age- and bone-age-dependent increases in SNR (*r* > 0.461, *p* ≤ 0.002) confirmed that FA trends reflected biological changes rather than technical artifacts. (3) Parameter optimization: A *b*-value of 600 s/mm^2^ and 25 diffusion gradients balanced FA stability and ADC precision, consistent with our prior validation (18). (4) ROI delineation was performed on planar echo images with *b* = 0 s/mm^2^ rather than T1WI images. This enabled the true delineation of the morphological scope of the cartilage, avoiding measurement errors caused by the deformation of DTI images (6, 13).

This study acknowledges several limitations. First, the exclusion of children under 6 years (due to poor compliance) and over 12 years (owing to thin cartilage affecting ROI delineation) limits the generalizability of the findings. Second, bone age assessment using the Chinese Wrist Bone Development Standard (CHN) method may be subject to observer subjectivity. Third, manual ROI delineation could introduce measurement errors. Finally, the study of a local cohort has limited the wide applicability of the data. Future studies should incorporate automated segmentation, expand age coverage, conduct multi-center research, and correlate DTI markers with histology to validate microstructural interpretations.

## 5 Conclusion

We establish normative FA and ADC values for knee cartilage in 6–12-year-old children, demonstrating their sensitivity to age-, bone age-, and sex-dependent developmental patterns. The stronger correlation of FA with maturation metrics, particularly bone age, supports its utility as a robust biomarker for non-invasive cartilage assessment, advancing the potential of quantitative MRI to monitor growth dynamics in pediatric populations.

.

## Data Availability

The raw data supporting the conclusions of this article will be made available by the authors, without undue reservation.
